# Human gut microbiota composition and its predicted functional properties in people with western and healthy dietary patterns

**DOI:** 10.1007/s00394-022-02928-6

**Published:** 2022-06-24

**Authors:** Anna M. Malinowska, Dieuwertje E. Kok, Wilma T. Steegenga, Guido J. E. J. Hooiveld, Agata Chmurzynska

**Affiliations:** 1 Department of Human Nutrition and Dietetics, Poznań University of Life Sciences, Wojska Polskiego 31, 60-624 Poznan, Poland; 2 Division of Human Nutrition and Health, Wageningen University and Research, PO Box 17, 6700 AA Wageningen, The Netherlands

**Keywords:** Western dietary pattern, Healthy dietary pattern, Gut microbiota, Stool transit time

## Abstract

**Purpose:**

Some dietary habits cluster together, and for this reason it is advised to study the impact of entire dietary patterns on human health, rather than that of individual dietary habits. The main objective of this study was to evaluate differences in gut microbiota composition and their predicted functional properties between people with a healthy (HDP) and western (WDP) dietary pattern.

**Methods:**

A cross-sectional, observational study was carried out on 200 participants enrolled 2017–2018 in Poznań, Poland, equally distributed into HDP and WDP groups. Diet was estimated using 3-day food records and information on stool transit times was collected. Fecal microbiota composition was assessed by 16S rRNA gene sequencing and its functional properties were predicted by the PICRUSt2 workflow.

**Results:**

The α-diversity did not differ between people with WDP and HDP, but β-diversity was associated with dietary pattern. People with HDP had higher relative abundances (RA) of Firmicutes and *Faecalibacterium* and lower RA of Bacteroidota and *Escherichia–Shigella* than participants with WDP. Only a small proportion of the variance in microbiota composition (1.8%) and its functional properties (2.9%) could be explained by dietary intake (legumes, simple sugars and their sources, like fruit, soft drinks) and stool transit characteristics.

**Conclusion:**

Gut microbiota composition and predicted metabolic potential is shaped by overall diet quality as well as the frequency of defecation; however, the cumulative effect of these explain only a relatively low proportion of variance.

**Supplementary Information:**

The online version contains supplementary material available at 10.1007/s00394-022-02928-6.

## Introduction

**Fig. 1:**
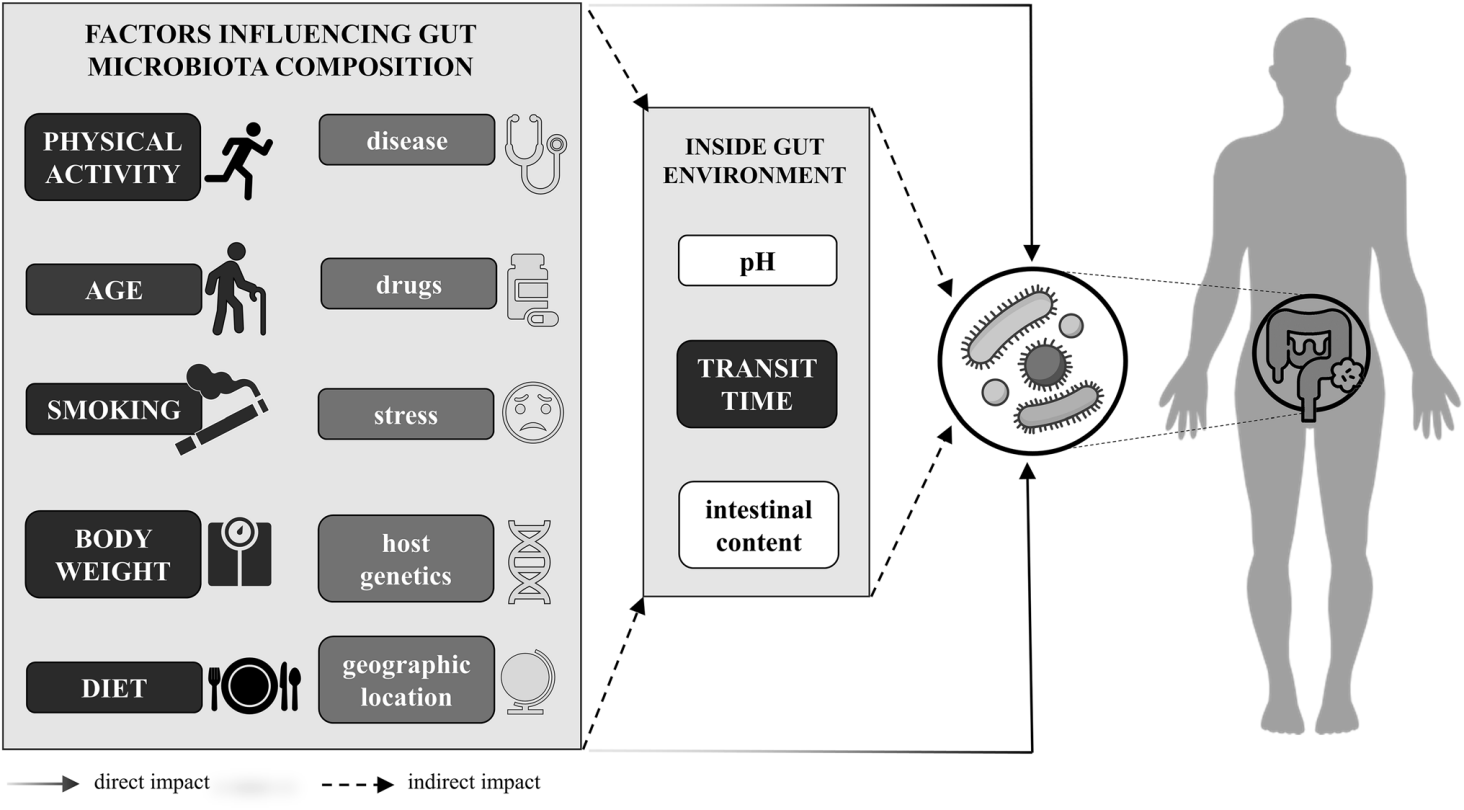
Factors influencing gut microbiota composition. Factors evaluated in this study are shown in capital letters on a black background

The gut microbiota composition is associated with the host’s health status [1] and it is affected by many factors in direct and indirect manners (Fig. 1). The indirect impact of some of those factors is manifested through the shaping of the intestinal environment. The conditions in the intestine depend, for example, on transit time [2] and intestinal content [3, 4]. Those factors may vary from person to person, depending on health state, drug use, dietary intake [5, 6], smoking habit [7], and physical activity [8–10]. All these factors are highly interrelated. Many factors explaining the variation in gut microbiota composition have been described, but most of the variation (over 80%) remains unexplained [11–13]. Knowing which factors contribute to the presence of health-related bacteria may enable the application of gut-microbiota-modulating strategies that promote better health. Such strategies may include dietary recommendations.

People do not eat individual nutrients, but instead consume foods combined as meals, consisting of a mixture of nutrients of different bioavailabilities. The bioavailability of these nutrients may differ by food matrix and interaction between food components [14]. Moreover, it has been shown that selected dietary habits usually cluster together so that, for example, people with high intakes of soda and sweet products tend to eat more snacks and fried potatoes [15]. For this reason, it is difficult to separate the impact of the intake of one food product or nutrient from another on health outcome in observational studies. Taking those facts into consideration, it has been suggested that nutrition research should focus on the impact of sets of eaten products, referred to as dietary patterns (DP), instead of the intake of individual nutrients on health outcomes [16, 17].

So far, the differences in gut microbiota composition between people consuming predefined dietary patterns (such as vegan/vegetarian or omnivore [18], or with different levels of adherence to the Mediterranean diet (MedDiet) [19–21]), have been investigated. However, studying people following a vegan/vegetarian diet does not imply that the quality of their diet is always much better than that of nonvegetarians, since the difference in Healthy Eating Index (HEI) for these diets has been reported to be relatively small (4.5%) in some studies [22]. In some other studies [23], dietary patterns are evaluated using data-driven approaches (e.g., principal component or factor analysis), although these derived dietary patterns may not reflect recommended eating habits and consequently the results may be more difficult to translate into dietary recommendations. Further research is needed to conclude whether a recommended healthy dietary pattern supports the presence of gut microbiota associated with health.

In most observational studies aimed at evaluating the associations between diet and microbiota, DP or diet quality of the study participants is not an inclusion or exclusion criterion, and consequently the variation in dietary intake and habits might not be large enough to detect such associations. Indeed, the diet quality of most general populations is generally rather moderate [24–26]. Considering this, we aimed to study people who have either a pronounced healthy or unhealthy dietary pattern, which can help to better understand differences in gut microbiota composition and microbial functional properties between these groups.

The main objective of this study was to evaluate the differences in gut microbiota composition and their predicted functional properties between people with very distinct DPs, namely the healthy and the western patterns. This approach ensured the high variability of dietary intake and enabled enrollment of fewer participants while still making it possible to find significant associations with DP. Furthermore, we aimed to determine which food product and food component intakes contribute to the differences in gut microbiota between the two groups. We aimed also to investigate whether those factors can explain gut microbiota composition and their predicted metabolic properties.

## Materials and methods

### Study design

A cross-sectional observational study was carried out in the Wielkopolska region. All procedures involving research with the study participants were approved by the Local Ethics Committee at Poznań University of Medical Sciences (permit number 486/2016). Since the primary outcome of this study was to detect the overall differences in gut microbiota composition between people with different dietary patterns, we calculated the sample size using the R *samplesize* package, assuming a 0.2 Shannon index difference between two groups and a standard deviation of mean value of 0.5 [27]. The resulting required sample size was one hundred participants in each group.

**Fig. 2:**
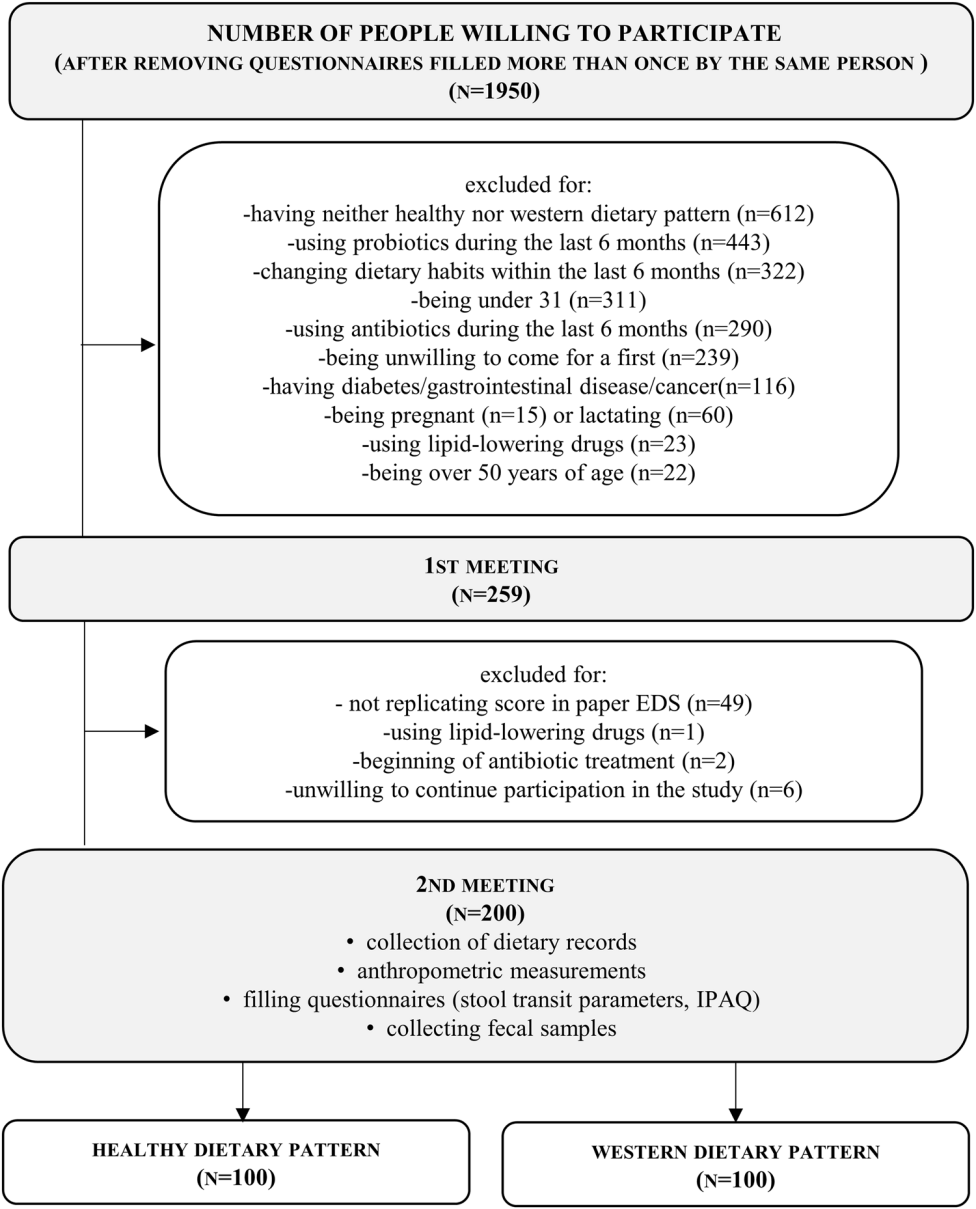
Study flow diagram (*n*, *N*—number of people)

Only adult participants, between 31 and 50 years of age, and having either healthy or western dietary pattern (determined twice using the Easy Diet Screener [28]) were enrolled in the study. A detailed description of the recruitment procedure has been given previously [28]. In brief, recruitment was conducted using online advertisements published using social media and paper flyers. In total, 1950 people were willing to participate and filled out the online questionnaire (Fig. 2), which asked about the dietary habits (EDS) and parameters described in the inclusion exclusion criteria. The reasons for excluding people from the study were: having neither a healthy nor western dietary pattern (34% of excluded people), using probiotics within last 6 months (23%), changing dietary habits within last 6 months (17%), being under 31 years of age (16%), using antibiotics within last 6 months (15%), being unwilling to come for a first meeting or to continue participation (13%), having diabetes, gastrointestinal disease, or cancer (6%), being pregnant or lactating (4%), using lipid-lowering drugs (1%), or being over 50 years old (1%). The percentages do not add to 100% because some of reasons for exclusion occurred simultaneously. Finally, a group of 200 adult participants, half of whom had the western dietary pattern (WDP) and the other half of whom had the healthy dietary pattern (HDP), with equal proportions of men and women, was enrolled between March and June 2017 as well as October 2017 and May 2018. Recruitment to each subgroup (male WDP, male HDP, female WDP, female HDP) finished as soon as there were 50 participants present. Eligible participants who gave written informed consent were invited to come in person to the Department of Human Nutrition and Dietetics, Poznań University of Life Sciences, where their anthropometric parameters were measured. The participants also contributed their dietary records from the previous week (or from 2 weeks before, where necessary) and stool samples. Each participant was earlier provided with a sample collection kit containing a paper stool collector (Kałszyk, Konrad Kosowski) and a sterile plastic tube (25 ml) with spoon attached to the lid. Participants were asked to fill at least half of the tube with stool sample and to keep it in a fridge for not longer than 24 h before coming to the University. DNA was immediately isolated from the chilled fecal samples.

### Dietary assessment

Participants were allocated to the WDP and HDP groups on the basis of the dietary assessment with the Easy Diet Screener (EDS) questionnaire [28], using cut-off score values of ≤ 14 or ≥ 21, respectively. We have shown previously that people allocated to WDP with EDS have worse diet quality and a higher risk of unfavorable lipid profiles, BMI values, and body compositions, than people in HDP [28]. To assess dietary intake of nutrients and food products participants were asked to record their intake for 3 consecutive days, one of which should have been a nonworking day. The nutritional value of the diets and the intake of food products was estimated using Diet 6.0 software (National Food and Nutrition Institute, Warsaw). Food products whose intake was evaluated are most commonly used to characterize HDP and WDP [16, 17]. To compensate for the varying energy intake of participants, associated with total intake of food, food intake was calculated as grams per 1000 kcal. Dietary fiber and salt intake were expressed in absolute values. Diet quality was assessed using EDS [28] and HEI [29, 30]. Briefly, in the case of EDS, 14 dietary habits for which the questions were asked in the screener were evaluated by scoring the answers 0, 1, or 2 and summing the score (to a maximum score of 28). Answers describing western dietary habits were scored 0, whereas those describing healthy dietary habits were scored 2. In case of HEI, that total score was calculated on the basis of the intake recorded in the dietary records. First, mixed foods were disaggregated using Diet 6.0 software (National Food and Nutrition Institute, Warsaw). Serving portions were then aligned with the US Department of Agriculture’s standard serving sizes, and finally the intakes of thirteen dietary components were evaluated by giving up to five or ten points for each component and adding to give the total score (to a maximum of 100).

### Determination of anthropometric parameters

Body weight and fat tissue content were determined using a BodPod air-displacement plethysmography system (Cosmed, USA) with the predicted thoracic gas volume. BMI was calculated as the body weight (kg) divided by height squared (m^2^). Waist and hip circumference were measured using non-stretchable tape and standard procedures and recorded to the nearest 1 mm. The waist-to-hip ratio (WHR) was calculated by dividing the waist circumference by the hip circumference.

### Determination of stool transit time parameters

During the first visit to the University, participants were asked to fill out a questionnaire concerning parameters that indirectly characterize stool transit time. They were asked to indicate the frequency of constipation, diarrhea, and sudden bowel movements and laxative use during last year. Each participant was additionally asked to indicate their number of bowel movements per week (open question) and their usual stool form, using the seven-point Bristol Stool Form Scale [31].

### Other parameters determination

Physical activity was described as low, medium, or high using the short form of the international physical activity questionnaire (IPAQ), which takes into account the number of days during week where vigorous, moderate-intensity activity, or walking takes place (for at least 20 or 30 min) [32]. Other questionnaire also asked participants whether they regularly or occasionally smoked traditional cigarettes, e-cigarettes, or did not smoke at all. Because of the substantial number of nonsmokers, regular and occasional users of traditional and e-cigarettes were grouped together and analyzed further as smokers; this variable thus only had two values: 0 for nonsmokers and 1 for smokers, regardless of the type of cigarette and the regularity of smoking.

### Gut microbiota composition analysis

DNA was extracted from fecal samples using the QIAamp DNA Stool Mini Kit (Qiagen). The isolated DNA was stored at + 4 °C until further use. Microbial community composition was assessed by 16S rRNA gene sequencing at an expected sequencing depth of 100 kb/sample. DNA encoding of the V3–V4 region of the 16S rRNA gene was amplified using the following primers: 341F (5’–CCTACGGGNGGCWGCAG–3’) and 785R (5’–GACTACHVGGGTATCTAATCC–3’) [33], generating amplicons of 444 bp. Q5 Hot Start High-Fidelity 2 × master mix was used in line with the manufacturer’s protocol. The amplicon mixture was sequenced (2 × 250 bp paired end) on a MiSeq System (Illumina; San Diego, CA, USA) with the use of v2 kit (Illumina), following the manufacturer’s protocol. Preliminary analysis was done on the MiSeq System with the use of MiSeq Reporter software v2.6. The analysis involved automatic demultiplexing of samples and generation of fastq files with raw reads.

To infer microbiota community composition, the amplicon sequence variant (ASV) method with the *dada2* Pipeline Workflow (1.8) was used [34]. In brief, the analysis proceeded as follows: (1) The adapter sequences were removed (cutadapt software [35]); (2) quality of reads analysis was performed and low quality sequences (those below 2, minimal length 50 bp) were removed; (3) identical reads were dereplicated; (4) the sample inference algorithm was applied; (5) paired reads were merged, chimeras were removed; (6) alignment to the reference database (SILVA v138) was performed, followed by species assignment [36]. A phylogenetic tree was constructed using the *phangorn* R package [37]. The contaminant and rare taxa were filtered by removing all taxa that are not assigned to any phylum. Only taxa with abundance over 0.25% in at least one sample were left in the dataset [38]. Then the RA was calculated once again on a filtered dataset.

The analysis of gut microbiota composition, yielding parameters such as relative abundance, α (the Shannon and inverse Simpson index) and β (PCoA using Bray–Curtis and weighted Unifrac distance on the ASV level) diversity, was performed using the *phyloseq* [39] and *microbiome* [40] packages. All analyses were performed using the relative abundance (RA) of taxons.

### Predicting the functional properties of gut microbiota

The functional properties of the microbiota community were predicted from amplicon sequences with the use of the Phylogenetic Investigation of Communities by Reconstruction of Unobserved States (PICRUSt2) software [41]. The predicted pathway abundances and coverages per sample, based on the predicted Enzyme Commission (EC) number abundances, were used for further analysis after calculating the relative abundance of pathways.

### Statistical analysis

The data were analyzed using R software version 4.0.4. The normality of data distribution was checked with the Shapiro–Wilk test. The statistical significances of the differences between people with WDP and HDP for normally distributed data without or after data transformation (energy intake [%EER]) were analyzed using Student’s *t*-test for unpaired samples, and the data were presented as means with standard deviations. In case of nonnormally distributed data, the Mann–Whitney *U*-test was used and the data were presented as medians with interquartile ranges (IQR). The p values of the Mann–Whitney *U*-test comparing the RA of phyla, genera, species, and predicted abundance of metabolic pathways were corrected using the FDR approach (*qvalue* package [42]) and the *q* values were then reported. For categorical data, a *χ*^2^ test was used. The base R functions and the *matrixTests* package were used in these analyses. Permutational analysis of variance (PERMANOVA) was used (the *vegan* package [43]) to test whether the bacterial composition and its predicted functional properties were related to DP.

To identify the most biologically informative features differentiating gut microbiota and their functional properties between people with HDP and WDP, the linear discriminant analysis (LDA) effect size (LEfSe) method was employed [44].

For all of the following analysis, the participants in the HDP and WDP groups were combined into one group to increase the variance in food and nutrient intake. The correlation analysis between the relative abundance of bacterial genera and nutritional, lifestyle habits, and parameters describing stool transit time were performed using Spearman correlation with the *Hmisc* package. *p* values were corrected for multiple testing [42]. The number of statistically significant correlations (with *p* value < 0.05) with the intake of food/nutrients considered healthy (recommended to be consumed more by World Health Organization and national nutritional guidelines for the general population [45–47], as well characteristic of HDP) and unhealthy (recommended to be avoided or limited by nutritional guidelines, as well characteristic of WDP) were calculated and presented as the percentage of the total amount of correlations considered. For those genera that had a considerable percentage of associations with healthy and unhealthy dietary habits (> 14%), and for genera that correlated with HEI, a correlation analysis with other lifestyle factors and stool transit parameters was performed.

Redundancy analysis (RDA) was used (the *vegan* package [43]) to test whether the microbiota composition (on the genus level) and its predicted functional properties might be explained by food intake, diet composition, lifestyle, or stool and bowel movement characteristics. To determine which of the explored sets of variables explained the greatest amount of variance and which dietary habits within each DP might be most meaningful in shaping gut microbiota composition and their functional properties we built four models. In Model 1, the intake of food products were used as the explanatory variables; in Model 2, the nutrients and HEI were used; while in Model 3, age, anthropometric, lifestyle parameters, and parameters describing stool transit time were used. In Model 4, all the explanatory variables from models 1–3 were used. Two variants were prepared for each of these models: a full model using all of the variables (multicollinearity was checked using the VIF/tolerance, with a threshold of ten, and excluding variables causing collinearity), and a stepwise-built model. Both the test factors and the relative abundances of gut microbiota or predicted pathways were scaled and centered.

**Fig. 3:**
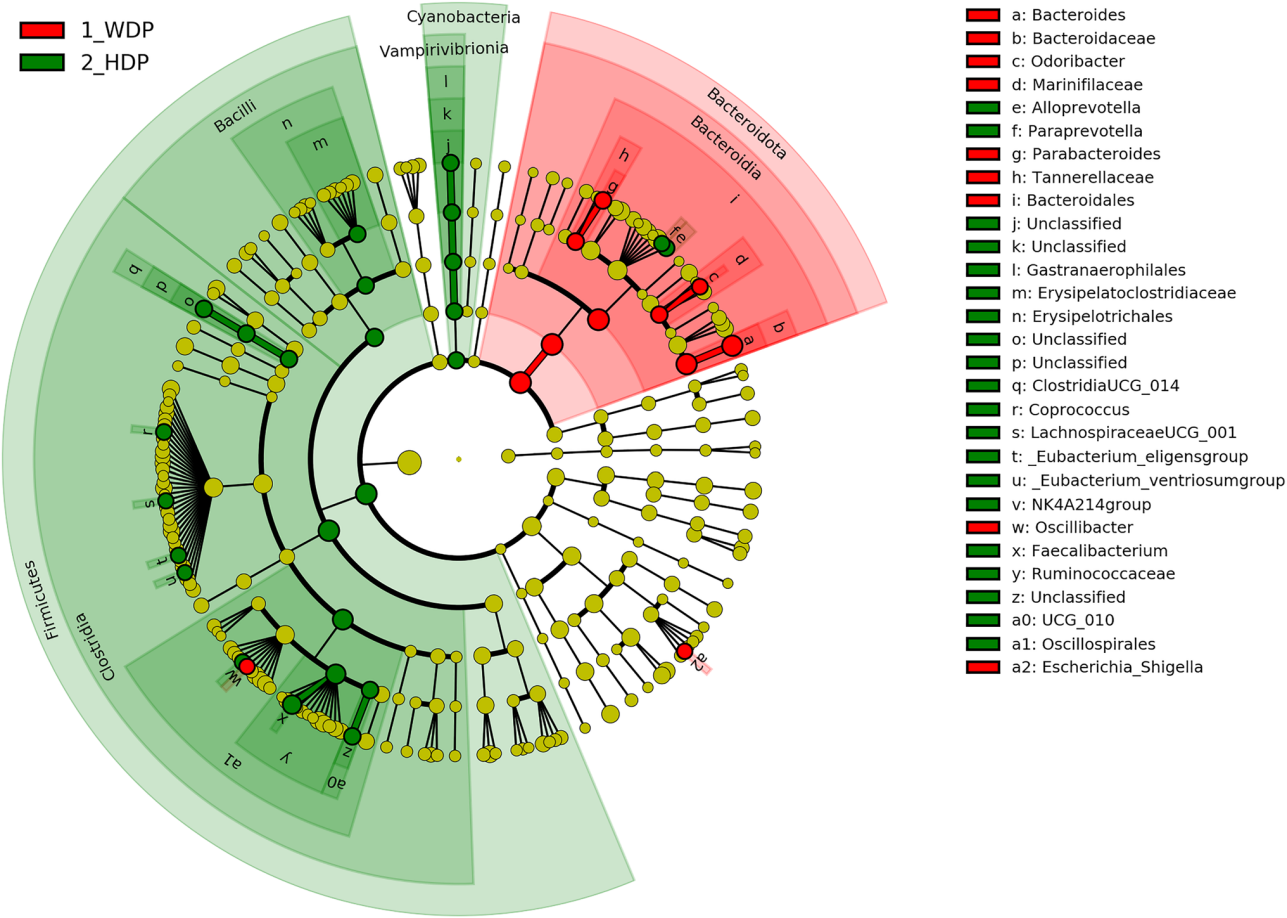
LEfSe results with threshold log_10_(LDA score) > 2 in the form of cladogram showing differences in the relative abundance of bacteria on all taxonomic levels between people with healthy and western dietary patterns; HDP: healthy dietary pattern; WDP: western dietary pattern

All the graphs other than Fig. 3 were prepared using the *ggplot2* package [48].

## Results

### Description of the study group

The study population consisted of 200 adult men and women, with a mean age of 38.2 ± 4.9 years. Other characteristics have been summarized in Table 1. In brief, people with WDP (*n* = 100) had higher BMI values, percentage of body fat, waist and hip circumferences, and WHR than did people with HDP (*n* = 100). The WDP group also contained statistically significantly more participants with low physical activity than the HDP group. Most participants did not smoke, and the number of smokers was independent of dietary pattern.

**Table 1:** Characteristics of participants with the western and healthy dietary patterns

Parameter	HDP	WDP	*p* (Mann–Whitney *U-*test, unless otherwise stated)
	Median ± IQR (unless otherwise stated)		
General characteristics			
Age [years]	38.6 ± 5.0^a^	37.8 ± 4.8^a^	0.215^c^
BMI [kg/m^2^]	24.0 ± 3.8^a^	25.6 ± 4.3^a^	0.008^c^
Body fat [%]	25.1 ± 9.0^a^	29.9 ± 9.2^a^	< 0.001^c^
Waist circumference [cm]	81.3 ± 19.3	87.0 ± 17.5	0.003
Hip circumference [cm]	91.5 ± 10.8	94.5 ± 12.0	0.011
WHR	0.89 ± 0.13	0.93 ± 0.13	0.006
IPAQ (low/medium/high)	11/40/49	24/41/35	0.028^d^
Smoking [Y/N]	14/85	18/81	0.563^d^
Parameters reflecting bowel transit time			
Bristol stool scale (≤ 2, 3 or 4, ≥ 5)	11/73/15^b^	15/63/17^b^	0.498^d^
Defecation [frequency/week]	10.5 ± 7.0	7.0 ± 4.5	0.003
Sudden bowel movement (never/rarely/sometimes/usually/always)	11/55/26/6/0^b^	6/45/35/10/0^b^	0.189^d^
Constipation (never/rarely/sometimes/usually/always)	37/4513/0/1^b^	29/35/20/10/1^b^	0.008^d^
Diarrhea (never/rarely/sometimes/usually/always)	23/58/15/1/0^b^	17/61/17/0/0^b^	0.556^d^
Laxative use (never/once per month/2–3 times per month/twice a week/most days)	95/2/2/0/0^b^	90/3/2/1/0^b^	0.732^d^

*BMI* Body Mass Index, *IPAQ* International Physical Activity Questionnaire, *Y/N* Yes/No

^a^Mean ± standard deviation

^b^Proportion of participants

^c^Student’s *t*- test

^d^*χ*^2^ test

The frequency of defecation was statistically significantly higher for participants with HDP than with WDP. People with a WDP declared that they suffered from constipation significantly more frequently (31% with WDP versus 14% with HDP suffered from constipation sometimes, usually, or always, *p* = 0.008). The rest of the parameters describing bowel transit time did not differ between the groups. The consistency of stool of most of the participants, whether with HDP or WDP, was normal (type 3 or 4 on the Bristol Stool Form Scale). Most participants (95% with HDP and 90% with WDP) did not use laxatives within the year prior to the study.

### Nutrient intake in HDP and WDP

Overall diet quality varied with mean HEI for participants with HDP, being 33% higher than for participants with WDP (76.9 ± 9.2 vs. 58.7 ± 10.5). The median intake of all food products considered unhealthy (e.g., animal fats, added sugar, salt, confectionery, savory snacks, etc.), other than refined cereals and groats, was significantly higher in participants with WDP than with HDP (Table 2). On the other hand, the intake of all products considered healthy (e.g., whole grains, fruits, vegetables, legumes, and nuts and seeds), other than fruit and vegetable juices, was significantly higher in participants with HDP than in participants with WDP. Participants with WDP had statistically significantly higher energy intake. People with HDP had significantly higher intake of dietary fiber (27.8 ± 14.4 vs. 19.6 ± 6.7 g/day, *p* < 0.001) and lower simple sugar intake (9.5 ± 5.6 vs. 11.7 ± 7.2%E, *p* = 0.017).

**Table 2:** Nutrient and food intake of participants with a western dietary pattern (WDP) and with healthy dietary pattern (HDP)

Parameter	HDP	WDP	*p* (Mann–Whitney *U-*test, unless otherwise stated)
	Median ± IQR (unless otherwise stated)		
Nutrient intake			
Energy [kcal]	2108 ± 835	2295 ± 833	0.002
Energy [%EER]	68.9 ± 17.0^a^	81.8 ± 23.9^a^	< 0.001^b^
Carbohydrates [%E]	49.4 ± 7.7^a^	48.0 ± 8.3^a^	0.233^b^
Simple carbohydrates [%E]	9.5 ± 5.6	11.7 ± 7.2	0.017
Fiber [g/d]	27.8 ± 14.4	19.6 ± 6.7	< 0.001
Protein [%E]	16.0 ± 4.2	13.7 ± 3.5	< 0.001
Fat [%E]	31.1 ± 7.3	32.8 ± 8.4	0.027
SFA [%E]	10.7 ± 5.4	12.7 ± 4.2	< 0.001
PUFA [%E]	5.5 ± 3.0	4.7 ± 2.2	< 0.001
Alcohol [%E]	0.7 ± 3.3	2.3 ± 5.8	0.006
Salt [g/d]	7.1 ± 2.8^a^	8.5 ± 2.7^a^	0.001^b^
Dietary pattern score and food groups intake			
EDS [score]	23 ± 3	11 ± 4	< 0.001
HEI [score]	76.9 ± 9.2^a^	58.7 ± 10.5^a^	< 0.001^b^
Refined bread [g/1000 kcal]	6.2 ± 22.6	37.8 ± 40.1	< 0.001
Wholegrain bread [g/1000 kcal]	21.7 ± 45.3	3.0 ± 23.7	< 0.001
Refined cereals, groats [g/1000 kcal]	0.0 ± 8.4	0.0 ± 3.5	0.060
Wholegrain cereals, groats [g/1000 kcal]	17.0 ± 28.5	0.0 ± 8.2	< 0.001
Plant fats [g/1000 kcal]	6.3 ± 5.9	4.0 ± 7.0	0.004
Animal fats [g/1000 kcal]	1.5 ± 4.8	5.9 ± 8.5	< 0.001
Low-fat dairy products [g/1000 kcal]^c^	67.5 ± 87.2	32.2 ± 46.3	< 0.001
High-fat dairy products [g/1000 kcal]^d^	10.6 ± 27.7	21.7 ± 39.6	< 0.001
Added sugar [g/1000 kcal]	0.7 ± 3.1	4.3 ± 8.1	< 0.001
Soft drinks [g/1000 kcal]	0.0 ± 0.0	0.0 ± 43.6	< 0.001
Confectionery [g/1000 kcal]	18.3 ± 30.0	28.8 ± 33.6	0.007
Savory snacks [g/1000 kcal]	0.0 ± 0.0	0.0 ± 0.1	0.034
Vegetables [g/1000 kcal]	160.0 ± 142.5	88.2 ± 66.7	< 0.001
Vegetable juice [g/1000 kcal]	0.0 ± 0.0	0.0 ± 0.0	0.157
Fruit [g/1000 kcal]	133.1 ± 109.6	56.1 ± 82.1	< 0.001
Fruit juice [g/1000 kcal]	0.3 ± 23.8	0.2 ± 36.9	0.771
Red meat [g/1000 kcal]	15.5 ± 29.9	28.9 ± 46.6	< 0.001
White meat and fish [g/1000 kcal]	26.7 ± 44.7	16.4 ± 29.5	0.022
Nuts and seeds [g/1000 kcal]	8.2 ± 12.7	1.8 ± 5.9	< 0.001
Legumes [g/1000 kcal]	2.7 ± 11.0	0.0 ± 0.7	< 0.001
Alcoholic beverages [portions^e^/day]	0.1 ± 0.3	0.2 ± 0.5	0.005

*IQR* interquartile range, *kcal* kilocalories, *EER* estimated energy requirement [49], *E* energy, *g/d* gram/day, *SFA* saturated fatty acids, *PUFA* polyunsaturated fatty acids, *EDS* Easy Diet Score, *HEI* Healthy Eating Index

^a^Mean ± standard deviation

^b^Student’s *t*-test

^c^Including low fat milk, yoghurts, cottage cheese

^d^Including high fat milk, cream, hard and soft cheese

^e^One portion taken as 330 ml of beer, 200 ml of wine, or 60 ml of high-alcohol drinks

### Dietary patterns and microbiota composition

The median number of reads per sample for the 16S rRNA gene amplicon dataset was 62,581 (range 20,152–122,175). In total, 2,069 different ASVs and 123 genera were identified. The fecal microbiota of participants belonging to either HDP or WDP was composed mainly of Bacteroidota (47.9% ± 15.9% vs. 51.4 ± 16.5%, respectively, *q* = 0.08), Firmicutes (41.4% ± 13.0% vs. 36.7% ± 19.3%, *q* = 0.08), Proteobacteria (5.6% ± 6.4% vs. 5.7% ± 7.4%, *q* = 0.70) and Verrucomicrobiota (0.79% ± 2.59% vs. 0.80 ± 2.80%, *q* = 0.70). The most pronounced statistically significant difference in the RA of genera between the groups (according to LEfSe analysis) was observed for *Bacteroides* and *Faecalibacterium*, with the first being more abundant in WDP and the second more abundant in HDP (Fig. 3, Table 3). Based on the LEfSe analysis, we found that the species that explain most of the differences between HDP and WDP, and which is characteristic of people with WDP, was *B. vulgatus* (RA of 12.60% ± 16.68% vs. 6.17% ± 14.98%, log_10_(LDA score) = 3.38), whereas the species most characteristic of people with HDP was *Faecalibacterium prausnitzii* (13.52% ± 12.43% vs. 7.51% ± 11.23%; log_10_(LDA score) = 3.10).

**Table 3:** Relative abundance of genera that differ between people with different dietary patterns

Phylum	Family	Genus	RA [%] median ± IQR		% of people with genus present (in HDP/WDP)	Delta in median [%]	Delta in mean [%]^*^	*q* value (Mann–Whitney *U*-test, corrected)	log_10_ (LDA score)
			HDP	WDP					
GENERA MORE ABUNDANT IN PEOPLE WITH HDP									
F	*Ruminococcaceae*	*Faecalibacterium*	6.58 ± 4.89	4.98 ± 4.58	99/99	1.61	1.50	0.02	3.10
	*Lachnospiraceae*	*Coprococcus*	0.36 ± 0.71	0.17 ± 0.38	97/90	0.18	0.32	0.01	2.36
	*Oscillospiraceae*	*NK4A214 group*	0.33 ± 0.48	0.18 ± 0.43	88/79	0.14	0.27	0.08	2.27
	*Lachnospiraceae*	*[Eubacterium] ventriosum group*	0.07 ± 0.11	0.12 ± 0.22	89/90	− 0.05	0.15	0.14	2.26
		*[Eubacterium] eligens group*	0.38 ± 0.57	0.18 ± 0.43	92/77	0.20	0.24	0.01	2.23
		*Lachnospiraceae UCG-001*	0.19 ± 0.49	0.09 ± 0.32	78/66	0.10	0.18	0.11	2.09
		*Blautia*	0.29 ± 0.4	0.22 ± 0.25	99/98	0.07	0.12	0.14	1.95
		*[Eubacterium] xylanophilum group*	0.27 ± 0.54	0.15 ± 0.49	89/70	0.12	0.07	0.11	1.89
		*Lachnospiraceae UCG-003*	0.00 ± 0.00	0.00 ± 0.00	18/7	0.00	0.11	0.12	1.87
B	*Prevotellaceae*	*Alloprevotella*	0.00 ± 0.00	0.00 ± 0.00	24/11	0.00	0.41	0.12	2.50
		*Paraprevotella*	0.00 ± 0.54	0.00 ± 0.18	45/27	0.00	0.04	0.14	2.01
GENERA MORE ABUNDANT IN PEOPLE WITH WDP									
B	*Bacteroidaceae*	*Bacteroides*	25.30 ± 24.44	32.16 ± 23.40	100/100	− 6.86	− 5.44	0.12	3.59
	*Tannerellaceae*	*Parabacteroides*	0.59 ± 1.17	0.95 ± 1.73	88/88	− 0.36	− 0.66	0.16	2.69
	*Marinifilaceae*	*Odoribacter*	0.43 ± 0.43	0.61 ± 0.52	98/95	− 0.18	− 0.16	0.03	2.06
P	*Enterobacteriaceae*	*Escherichia-Shigella*	0.00 ± 0.07	0.03 ± 0.15	44/67	− 0.03	− 0.33	0.03	2.32
F	*Oscillospiraceae*	*Oscillibacter*	0.19 ± 0.31	0.34 ± 0.49	97/96	− 0.15	− 0.18	0.01	2.15
	*Lachnospiraceae*	*Lachnoclostridium*	0.18 ± 0.22	0.21 ± 0.28	95/98	− 0.03	− 0.10	0.20	1.89
	*Lachnospiraceae*	*[Ruminococcus] torques group*	0.10 ± 0.25	0.16 ± 0.38	82/87	− 0.06	− 0.08	0.16	1.83
	*Oscillospiraceae*	*Flavonifractor*	0.00 ± 0.04	0.05 ± 0.1	38/65	− 0.05	− 0.04	0.01	1.47
	*Ruminococcaceae*	*UBA1819*	0.00 ± 0.02	0.02 ± 0.05	47/72	− 0.02	0.00	0.01	1.40
	*Lachnospiraceae*	*Tuzzerella*	0.00 ± 0.00	0.00 ± 0.00	3/14	0.00	− 0.02	0.07	1.15
	*Ruminococcaceae*	*Negativibacillus*	0.00 ± 0.00	0.00 ± 0.00	3/14	0.00	− 0.01	0.07	0.94

Only those genera for which *q* < 0.1 or log_10_(LDA score) > 1.8 are presented. Genus has been sorted by log_10_(LDA score) in descending order within each phylum

*RA* relative abundance, *HDP* healthy dietary pattern, *WDP* western dietary pattern, *B* Bacteroidota, *F* Firmicutes, *P* Proteobacteria

^*^The difference in means is also presented, because in some cases both medians are 0.00

**Fig. 4:**
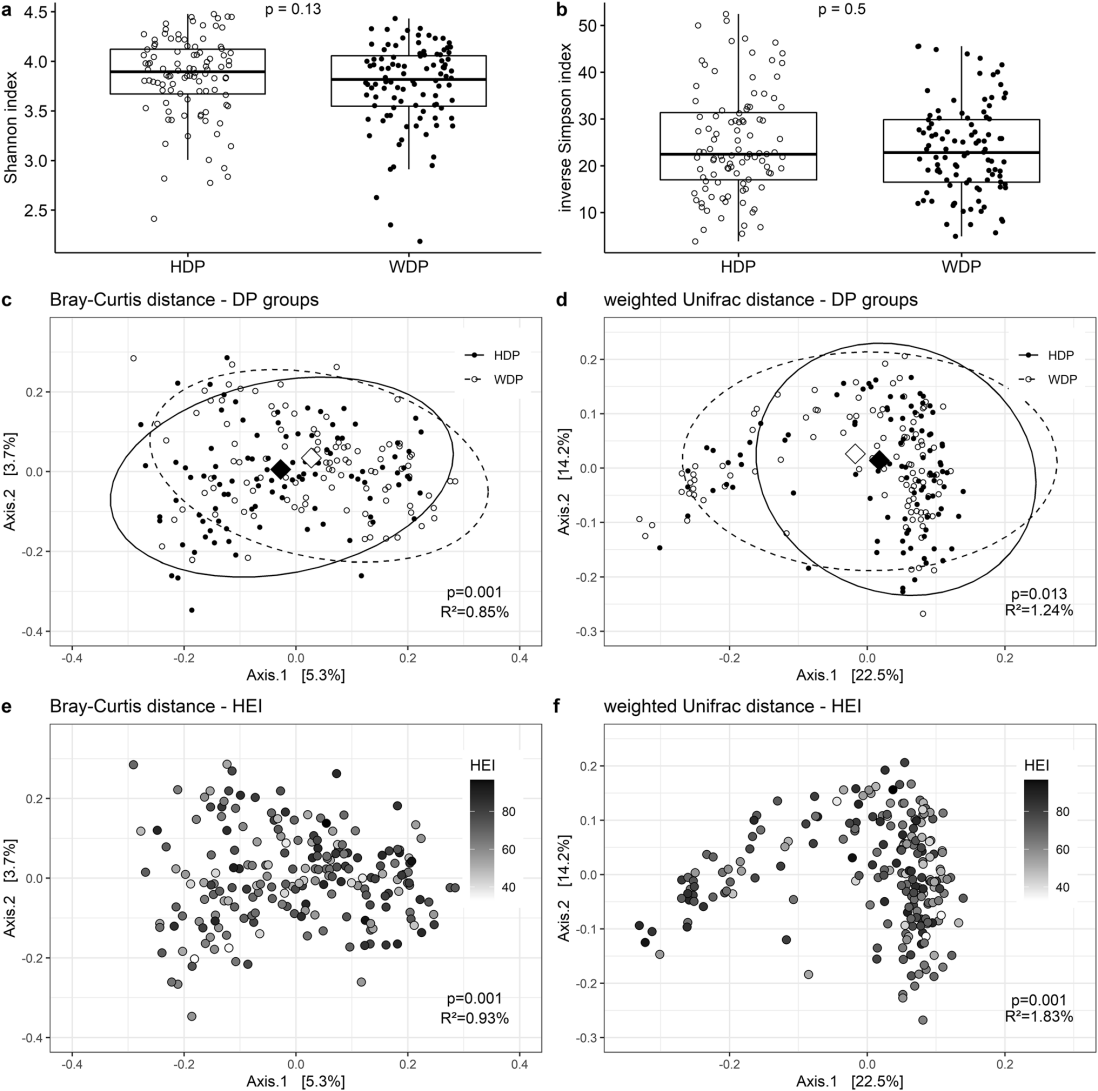
Plots illustrating bacteria diversity in the study group: α-diversity showing Shannon (**a**) richness and inverse Simpson index (**b**); β-diversity showing PCoA plots (centroids marked with diamonds) of Bray–Curtis (**c**, **e**) and weighted Unifrac (**d**, **f**) distances on the ASV level. The results of statistical analysis have been shown on each plot (Mann–Whitney *U*-test for **a** and **b**, and PERMANOVA for **c**, **d**, **e** and **f**). Plots **c** and **d** show associations with the DP groups, whereas plots e and f show associations with diet quality, as measured by HEI

Although the mean number of observed ASVs was statistically significantly higher in participants with HDP compared to those with WDP (167 ± 52 vs. 157 ± 45, *p* = 0.019), the indices of α-diversity—namely the Shannon index (3.90 ± 0.45 vs. 3.82 ± 0.51, *p* = 0.13) and the inverse Simpson index (22.5 ± 14.4 vs. 22.8 ± 13.4, *p* = 0.50)—did not differ statistically significantly between the groups (Fig. 4a, b).

β-diversity was associated with dietary pattern, regardless of whether a phylogenetic-tree dependent (weighted Unifrac) or independent (Bray–Curtis) (Fig. 4c) distance measure was used (Fig. 4d). PCoA ordination plots showed that the centroids of each ellipsis were located differently in case of Bray–Curtis distance (*p* = 0.001) but in case of weighted Unifrac the position of centroids was similar (*p* = 0.057); the dispersion was similar, but the distinction between two groups was not very clear. The coefficient of determination (*R*^2^) indicated that the variance in the microbiota composition could be explained by two distinct DPs in 0.85%, in the case of Bray–Curtis distance, or 1.24% in case of the weighted Unifrac distance. Models adjusted for BMI and physical activity were still statistically significant, with *R*^2^ of 2.1% (*p* = 0.001) or 2.2% (*p* = 0.034) in case of Bray–Curtis or weighted Unifrac distance, respectively. Moreover, when HEI measure was used in the PERMANOVA analysis, in place of the DP group classifications, the models were still statistically significant (*R*^2^ = 0.93%, *p* = 0.001 and *R*^2^ = 1.83%, *p* = 0.001, respectively) (Fig. 4e, f) also after adjustment for BMI and physical activity (*R*^2^ = 2.2%, *p* = 0.001 and *R*^2^ = 2.7%, *p* = 0.009, respectively.)

A Spearman correlation test was conducted to observe which nutritional factors contributed to the association with DP and diet quality. Bacteria whose RA correlated mostly with healthy dietary habits (Online Supplementary Resource 1 and 2) predominantly correlated positively with the intake of fiber, vegetables, fruits, wholegrain cereal and nuts and seeds, and correlated negatively with the intake of refined bread, red meat and soft drinks. For *Lachnospiraceae UCG-001*, correlations with the intake of nuts and seeds remained significant after FDR correction (*r* = 0.35, *q* = 0.001). Such a situation was also seen in the case of *Faecalibacterium* and the intake of fruits (*r* = 0.30, *q* = 0.014) (Online Supplementary Resource 3). The bacteria showing correlations with healthy dietary habits included most of those that were more abundant in HDP (Table 3). Four bacteria genera (*UBA1819*, *Flavonifractor*, *Oscillibacter, Escherichia–Shigella*) with the highest number of correlations with unhealthy dietary habits (mainly with the intake of soft drinks, added sugar and high-fat dairy), were also negatively associated with HEI (*r* = 0.33, *q* = 0.002, *r* = 0.28; *q* = 0.04; *r* = 0.28, *q* = 0.04; *r* = − 0.30, *q* = 0.015, respectively) (Online Supplementary Resource 3).

### Comparison of functional properties of gut microbiota between participants with different dietary patterns

**Fig. 5:**
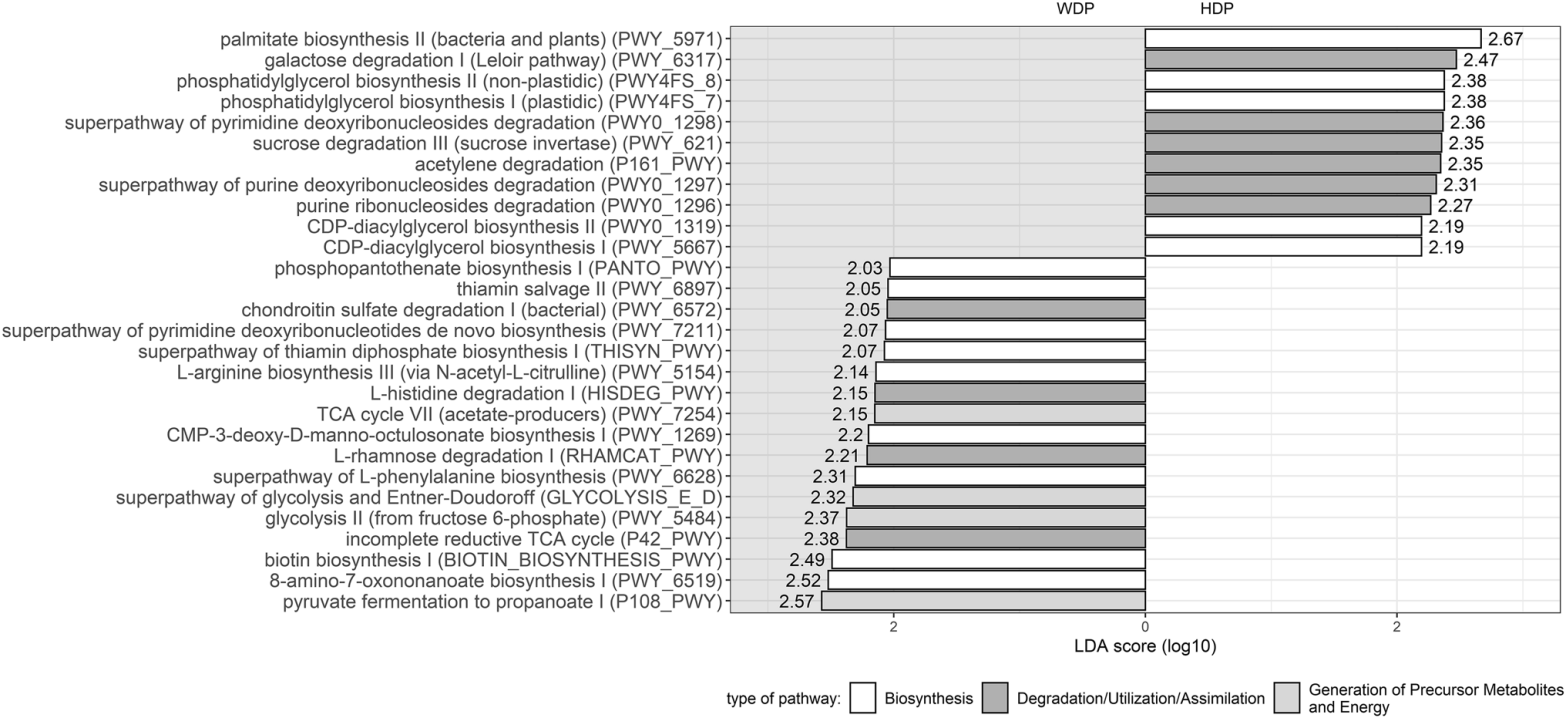
Results of LEfSe with threshold log_10_(LDA score) > 2 showing differences in the relative abundance of predicted metabolic pathways between people with healthy (HDP) and western (WDP) dietary patterns; pathways more abundant in people with HDP have been plotted on a white background, whereas those more abundant in people with WDP have been plotted on a grey background

To check whether differences in gut microbiota composition are translated into differences in pathway abundance, analysis of the predicted functional properties was performed. LEfSe analysis showed that the predicted functional properties of the microbiota were differentially represented across the dietary pattern groups (Fig. 5), being more abundant in people with the WDP pathway for propionate synthesis from pyruvate (P108-PWY, 0.56 ± 0.16% vs. 0.50 ± 0.21%), for biosynthesis of vitamins like biotin (BIOTIN-BIOSYNTHESIS-PWY, 0.42 ± 0.21% vs. 0.38 ± 0.16%) and thiamin (THISYN-PWY, 0.61 ± 0.08% vs. 0.58 ± 0.08%), and also for synthesis of CMP- ketodeoxyoctonate (Kdo) (PWY-1269, 0.45 ± 0.14% vs. 0.42 ± 0.12%). On the other hand, the gut microbiota of people with HDP had higher RA of pathways for synthesizing palmitate (PWY-5971, 0.34 ± 0.21% vs. 0.23 ± 0.30%), phosphatidylglycerol (PWY4FS-7 and PWY4FS-8, 0.68 ± 0.10% vs. 0.63 ± 0.16%), and CDP-diacylglycerol (PWY0-1319 and PWY-5667, 0.87 ± 0.12% vs. 0.83 ± 0.13%), and for degrading sucrose (PWY-621, 0.33 ± 0.12% vs. 0.29 ± 0.12%) and acetylene (P161-PWY, 0.37 ± 0.18% vs. 0.31 ± 0.20%).

PERMANOVA analysis showed that the overall profile of the predicted functional properties was affected by the DP when either DP categories (*p* = 0.039, *R*^2^ = 1.10%) or gradually increasing indices of HEI (*p* = 0.045, *R*^2^ = 1.14%) were used in the analysis.

### The effects of nutritional and lifestyle factors on microbiota composition

**Fig. 6:**
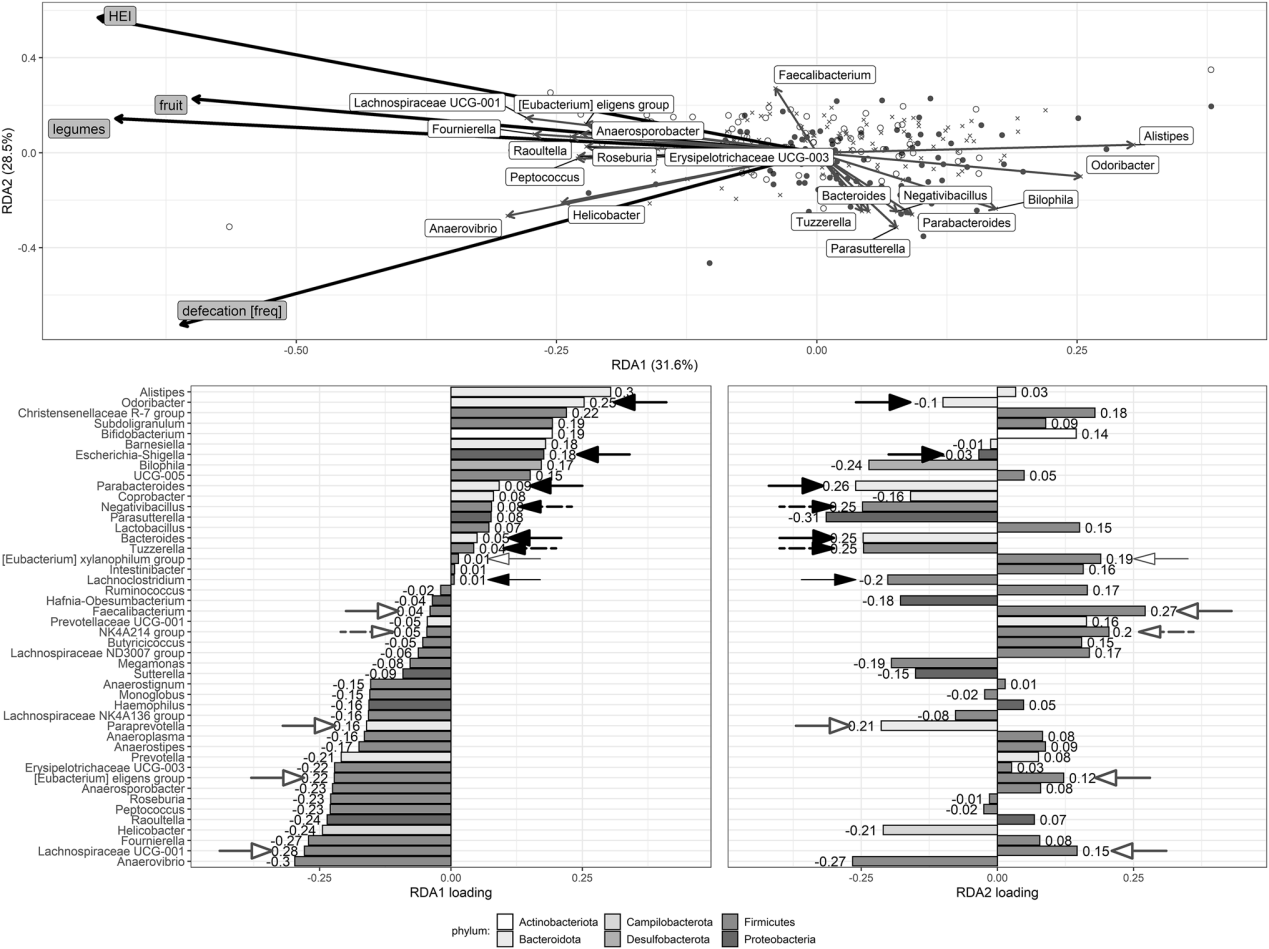
RDA plots showing factors that significantly explain the variance in the microbiota composition and the loadings for each component of RDA1 and RDA2 (stepwise-built Model 4 shown in Supplementary resource 4). Study participants’ weighted scores are represented by black-filled circles (WDP) and empty circles (HDP). Crosses show scores for genera. Thick black arrows represent explanatory variables, while thin grey arrows represent genera. Genera that were weakly associated with the first two axes are omitted for clarity. Loadings for RDA1 and RDA2 axes are shown below. Bars for the genera with significantly higher relative abundance in people with WDP and HDP (LEfSe result) have been indicated with black-filled and white-filled arrows, respectively (thicker arrows correspond to bacteria where log_10_(LDA score) > 2.0, thinner lines to bacteria where 1.8 < log_10_(LDA score) < 2.0, whereas dashed arrows to bacteria differentially abundant shown only in Mann–Whitney *U*-test)

In the RDA models that were statistically significant, only a small amount of the variation in microbiota composition (1.0–3.9%) was redundant with the variation in the explanatory variables (Online Supplementary Resource 4). RDA analysis showed that the variations observed in the relative abundances of genera cannot be explained by the entire set of food products (Model 1) or by the intake of food components (Model 2). However, the stepwise-built models included the intakes of legumes, fruit and of low-fat dairy products in Model 1 and HEI in Model 2. These models explained 1.04% and 0.50% of observed variance in microbiota composition, respectively. In Model 3, anthropometric, lifestyle, and stool transit time data were used as explanatory variables, and statistical significance was obtained (*p* = 0.001) only in stepwise-built Model 3 (not the full one); this explained 0.76% of the variance. In this model, two variables were selected: frequency of defecation and Bristol Stool Form Scale. Model 4 included all variables from the previous models and was built stepwise; this obtained statistical significance (*p* = 0.001) and explained 1.77% of variance. In this model, several nutritional factors were included (intake of legumes, fruit, and HEI), but so was the frequency of defecation (Fig. 6). For this model, the two constrained axes proved statistically significant (*p* < 0.01).

The RDA plot in Fig. 6 does not show a clear clustering of people with HDP and WDP. A higher legume and fruit intake (RDA1 axis) were associated with genera such as *Anaerovibrio, Fournierella*, *Helicobacter Raoultella* but also with some of the bacterial genera found in higher abundance in people with HDP. On the other hand, lower values of these variables were associated with the presence of *Allistipes*, *Christensellaceae R-7* group*, Subdoligranulum*, *Bifidobacterium* and *Barnesiella*, but also with some of the bacterial genera found in higher abundance in people with WDP. Furthermore, higher HEI score and lower frequency of defecation (RDA2 axis) were associated with a higher abundance of some of the bacteria found more commonly in the HDP group, and with a lower abundance of some of the bacteria observed in higher RA in the WDP group. The RA of the rest of the bacterial genera explained by this model did not differ between those with HDP and those with WDP (Table 3).

### Effects of nutritional and lifestyle factors on variance in the predicted functional properties of microbiota

As in the RDA models explaining the associations between gut microbiota composition and various explanatory parameters, of all the statistically significant built RDA models explaining variance in the predicted functional properties of the microbiota, only a small part of the variation (1.4–6.0%) was redundant with the variation in the explanatory variables (Online Supplementary Resource 5). In the stepwise-built models, the variables selected depended on the model: intake of added sugar, legumes and portions of alcohol in Model 1; simple sugar, SFA intake and HEI in Model 2; the frequency of defecation in Model 3. In all the stepwise-built models, the constrained axis 1 was statistically significant, but Model 1 explained greatest amount of variance (the adjusted *R*^2^ was 1.59%, compared to 1.24% and 0.91% in two remaining models). Model 4, which was built in a stepwise manner and included all the explanatory variables from the other models, was statistically significant (*p* = 0.001) and explained 2.91% of the variance. For this model, the first constrained axis, was statistically significant (*p* = 0.001).

**Fig. 7:**
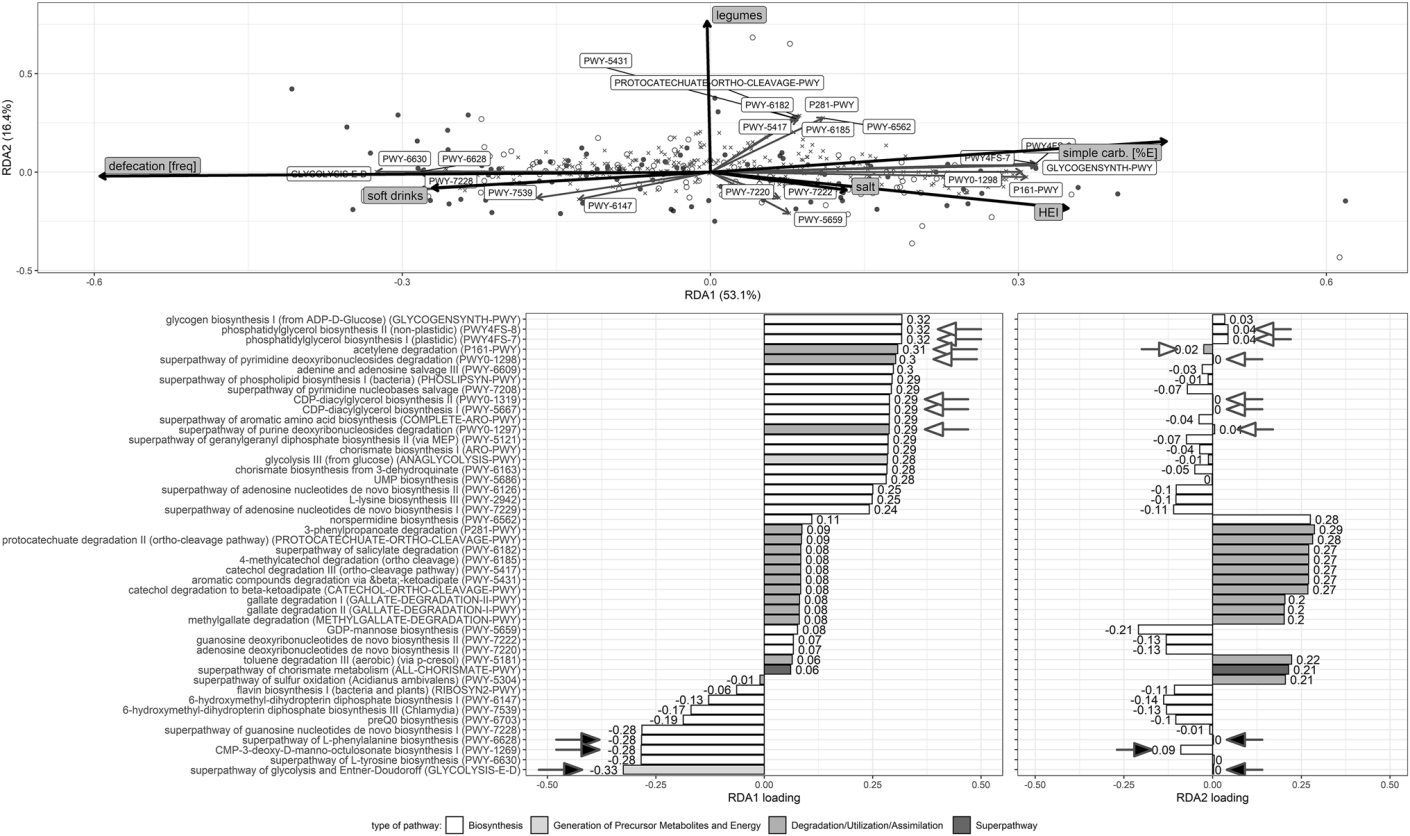
RDA plots showing factors that significantly explain the variance in the predicted metabolic pathways’ diversity and loadings for each component of RDA1 and RDA2 (stepwise-built Model 4 shown in Supplementary resource 5). Study participants weighted scores are represented by black-filled (WDP) and empty (HDP) circles. Crosses show scores for pathways. Thick black arrows represent explanatory variables, while thin grey arrows represent pathways. Pathways that were weakly associated with the first two axes were omitted for clarity. Loadings for RDA1 and RDA2 axes are shown below. Bars for the pathways with significantly higher relative abundance in people with WDP and HDP (LEfSe result) have been indicated with black-filled and white-filled arrows, respectively (where log_10_(LDA score) > 2.0)

The RDA plot in Fig. 7 shows that a higher frequency of defecation and higher soft drinks intake is positively associated with the synthesis of Kdo (PWY-1269), preQ_0_ (PWY-6703) and pterin (PWY-6147, PWY-7539). On the other hand, lower values of these factors, as well as higher intake of simple carbohydrates and salt and higher HEI, are associated with acetate (P161-PWY) and geranylgeranyl diphosphate (PWY-5121) production, and with synthesis of membrane components like phospholipids (PHOSLIPSYN-PWY, PWY-5667, PWY0-1319). Some of those pathways are also found in higher abundance in the gut microbiota of people with HDP (Fig. 5).

## Discussion

This study has shown that allocation to DP may explain only small proportion of variance (~ 1%) in gut microbiota composition and in functional properties of fecal microbiota. However, when also the impact of other factors—such as anthropometric parameters, specific nutritional habits and intakes, and stool transit time—are taken in consideration, 1.8% (Online Supplementary Resource 4) of the variance in gut microbiota composition and 2.9% of the variance in their predicted functional properties (Online Supplementary Resource 5) can be explained. Moreover, we showed that, apart from dietary pattern, some specific dietary habits and intakes—like intake of legumes or simple sugars and their sources (fruit, soft drinks)—may play a role in shaping fecal microbiota composition and its functional properties. The non-nutritional factor most pronouncedly associated with both gut microbiota composition and its functional properties was the frequency of defecation. Previous studies reporting potential factors affecting gut microbiota composition have shown that the combined effect of various health and lifestyle-related variables in explaining variance in microbiota composition accounts for ~ 16–20% of variance [11–13]. Moreover, the cumulative effect of these variables accounted for 7.7% [11]. One of the most important factors explaining gut microbiota variance in the studies turned out to be medication use, the intake of fruits, vegetables, bread, beverages, alcohol, total intake of carbohydrates, as well as Bristol Stool score [11–13]. Our results are in line with these, as we showed that frequency of defecation, which is associated with stool consistency, is a determinant of gut microbiota composition. It is worth noticing that the frequency of defecation might result from the composition of the diet as well as from other factors, including health status, medication use, physical activity, etc. (Fig. 1). However, we also showed that nutritional factors play an independent role of similar importance to that of stool transit time in shaping gut microbiota composition and their predicted functional profile. Although we studied a smaller group of people than did previous studies, the two dietary patterns were quite distinct (Table 2), which increased the chances of finding differences in gut microbiota between the dietary pattern groups.

Similarly to other studies [20, 23, 50, 51], we showed higher RA for two genera from *Prevotellaceae* family, namely *Alloprevotella* and *Paraprevotella* in individuals with a healthy dietary pattern. The last genus was also shown in the study of Shikany et al. [23] as being associated with prudent DP. Like the Nu-AGe MedDiet 12-month intervention [50] and PREDICT 1 study [52], we showed that HDP is associated with higher RA in *Faecalibacterium prausnitzii* and *[Eubacterium] eligens* group, and that WDP is associated with *[Ruminococcus] torques* group and *Flavonifractor.*

*Faecalibacterium prausnitzii*, found in higher abundance in people with HDP, seems to have positive impact on human health, since an inverse association between its RA and bowel disease, diabetes, and colorectal cancer has been found [53]. Furthermore, *Eubacterium eligens*, also found in the HDP group, has been shown to be a fiber-degrader and a producer of anti-inflammatory IL-10 [54]. On the other hand, bacteria found in people with WDP seem to have negative impact on health. For example, *Ruminococcus torques* species have been associated with conditions such as inflammatory bowel disease (IBD), vascular and inflammatory diseases, chronic kidney disease, and peripheral neuropathy, associated with type-2 diabetes [55, 56]. Moreover, *Shigella* is a pathogen that causes diarrhea [57] and *Escherichia–Shigella* have been found in higher abundance in people with prediabetes [58] and with diabetic peripheral neuropathy [55]. It should be noted that although an association between gut microbiota composition and many diseases has been shown in various studies, the results are sometimes contradictory and difficult to replicate [52]. This implies difficulties in the interpretation of results. Moreover functional properties seem more highly conserved across samples than across taxa, suggesting that although differences in gut microbiota composition might be observed across samples, this need not be translated into differences in the functional properties of gut microbiota [59]. We thus also aimed to investigate the association between the predicted functional properties of the fecal microbiota community and several lifestyle variables. We showed that, apart from the overall diet composition, there are other factors that might explain the variability in the functional properties of gut microbiota, such as frequency of defecation, but also some specific dietary intake (simple sugars, legumes, salt, and soft drinks). It is difficult to state which of these factors lead to an overall more positive health outcomes. On one hand, greater soft drinks intake, and more frequent defecation are associated with such positive outcomes, like the synthesis of pterin (precursor for the biosynthesis of several important cofactors, including tetrahydrofolate [60]) and preQ_0_ (metabolite with anti-cancer activity [61]). On the other hand, those factors are also associated with the synthesis of Kdo, which is a component of bacterial endotoxin, namely lipopolysaccharide (LPS) [62]—a pathogenic element related to the establishment and progression of intestinal inflammatory disorders [63].

One factor that partly explained the variation in pathway composition, as well as microbiota composition, was frequency of defecation. Interestingly, high defecation frequency explained the presence of those bacteria that were more abundant in HDP, but also some of the pathways that were less abundant in this DP. This result confirms the existence of redundancy in the functional properties of gut microbiota [64]. It moreover suggests that there exists another underlying factor that may explain the variance in both gut microbiota composition and its predicted pathways, since the presence of other pathways and taxa that were not typical of WDP or HDP can be explained by frequency of defecation.

One strength of the study is that the age range of all participants was relatively small (20 years) and the studied DPs were either considered rather healthy or unhealthy, which increased the variability in the food intake. However, this was an observational study which cannot demonstrate causal relationships. Participants in different DP groups also differed in terms of other variables, such as body weight, physical activity (though the models remained statistically significant after adjusting for these factors in PERMANOVA), and probably other confounding factors that were not taken into account in this study.

Although this study expands our knowledge of the association between nutrition and gut microbiota composition and their nutritional properties, it has several limitations. Dietary intake was evaluated on the basis of 3-day diet records, which may not represent the long-term dietary habits of participants (although those people who had recently changed their DP were excluded from the study) and some participants might have changed their diet during the recording period. Apart from that, we did not include socio-economic status, medical history, mood of participants, or other factors that could also affect gut microbiota composition, or their functional properties. Moreover, the functional properties of gut microbiota were predicted from ASV sequences. This analysis shows the functional potential of gut microbiota, but does not necessarily confirm that these pathways are active in this gut microbiota community, since they do not need to be expressed by the bacteria. Moreover, it is difficult to predict the metabolites produced by bacteria, since it does not take the interactions between the produced metabolites and cross-feeding between bacteria into account [59]. In the future studies, it is thus recommended to study the functional properties of gut microbiome on the basis of their transcriptome, their proteome, or by running ex vivo studies with culturing fecal material. Additionally, intervention studies are advised for demonstrating the causal impact of dietary pattern on gut microbiota composition or its functional properties. This would allow results to be better translated into dietary recommendations.

In conclusion, we showed gut microbiota composition and predicted function might differ between healthy and unhealthy dietary patterns and that they are also shaped by the frequency of defecation. The abundance of potentially favorable gut microbiota is mainly associated with a high intake of vegetables, fruits and fiber, whereas the abundance of the suggested unfavorable gut microbiota is mainly associated with the high intake of added sugar and soft drinks and the low intake of fiber. Although allocation to DP is associated with the diversity in gut microbiota composition and in its functional properties, it seems that the level of adherence to dietary recommendations (continuous measurement of HEI) and several specific dietary habits, like intake of legumes, sugar, and sources of sugar (soft drinks, fruits), may be of greater importance in explaining the variability in fecal microbiota composition and their predicted functional properties.

## Supplementary Information

Below is the link to the electronic supplementary material.

Supplementary file1 Supplementary Resource 1. Heatmaps showing the correlation between the relative abundances of a selected microbiota genus (from the Firmicutes phylum), dietary pattern scores, intake of chosen food products and macronutrients, as well as anthropometric measurements and parameters characterizing stool transit time in the group. Healthy eating habits have been surrounded on the heatmap by a square with thick lines. Spearman correlation coefficients are given only when p <0.05. Those marked with ** have q values < 0.05, while those marked with * have q values < 0.1 and > 0.05, whereas those without stars have a q value > 0.1 and a p value < 0.05. Only those bacteria genera which correlated significantly (q value < 0.05) with one of the dietary parameters or correlated with p value < 0.05 with HEI, or at least four healthy or unhealthy eating habits, are shown. Each column on the heatmap corresponds to the same bacterial genus as the bar plot shown on the bottom. The bar plot shows the total percentage of healthy and unhealthy correlations with p value < 0.05. Bacteria from the Clostridia class are marked in purple/violet on a bar plot, while those from other classes are marked in blue (TIFF 215378 KB)

Supplementary file2 Supplementary Resource 2. Heatmaps showing the correlation between the relative abundance of selected microbiota genera (from phyla other than Firmicutes), dietary pattern scores, intake of selected food products and macronutrients, as well as anthropometric measurements and parameters characterizing stool transit time in the analyzed group. Healthy eating habits have been surrounded on the heatmap by a square with thick lines. Spearman correlation coefficients are given only when p <0.05. Those marked with ** have q values < 0.05, whereas those without stars have q value > 0.1 and p value < 0.05. Only those bacteria genera that correlated significantly (q value < 0.05) with one of the dietary parameters or correlated with a p value < 0.05 with HEI, or with at least four healthy or unhealthy eating habits, are shown. Each column on the heatmap corresponds to the same bacterial genus as the bar plot shown on the bottom. The bar plot shows total percentage of healthy and unhealthy correlations with p value < 0.05. Bacteria from the Proteobacteria phylum are marked in red and orange on a bar plot, while those from the Bacteroidota phylum are marked in green; all other phyla are marked in yellow (TIFF 143069 KB)

Supplementary file3 Supplementary Resource 3. Scatter plots showing the association between the RA of selected bacteria genera and dietary intakes for which q < 0.05: (a) the correlation between RA of UBA1819 and HEI, (b) the correlation between the RA of Escherichia-Shigella and HEI, (c) the correlation between the RA of Oscillibacter and HEI, (d) the correlation between the RA of Flavonifractor and HEI, (e) the correlation between RA of Lachnospiraceae UCG-001 and the intake of nuts and seeds, and (f) the correlation between RA of Faecalibacterium and fruit intake (TIFF 88413 KB)

Supplementary file4 (PDF 85 KB)

Supplementary file5 (PDF 107 KB)
