# Supplementary material for: Human gut microbiota composition and its predicted functional properties in people with western and healthy dietary patterns
Source: Eur J Nutr. 2022 Jun 24;61(8):3887–903. doi: 10.1007/s00394-022-02928-6 (PMC9596542; doi:10.1007/s00394-022-02928-6)
Supplement: Supplementary file 4 — Supplementary file4 (PDF 85 KB) [file 394_2022_2928_MOESM4_ESM.pdf]

Supplementary Resource 4. RDA models explaining the variance in microbiota. The results of statistical analysis for the tested factors correspond to marginal term analysis in the model including all other variables.

| | Model 1, using food products[1] | | | | Model 2, using food components and HEI[2] | | | | Model 3, using anthropometric, lifestyle, and stool transit time data[3] | | | | Model 4, using all variables from the other analyses[4] | | | |
|---|---|---|---|---|---|---|---|---|---|---|---|---|---|---|---|---|
| parameters of the model | | variance [%] | F | p | | variance [%] | F | p | | variance [%] | F | p | | variance [%] | F | p |
| RDA1 | | 1.52 | 2.45 | 0.298 | | 1.48 | 2.40 | 0.005 | | 1.67 | 2.57 | 0.012 | | 2.31 | 3.56 | 0.158 |
| RDA2 | | 1.29 | 2.08 | 0.724 | | 0.88 | 1.43 | 0.812 | | 1.02 | 1.57 | 0.84 | | 2.19 | 3.38 | 0.201 |
| p (whole model) | 0.496 | | | | 0.071 | | | | 0.159 | | | | 0.257 | | | |
| $R^2$ (adj.) [%] | -0.02 | | | | 0.40 | | | | 0.37 | | | | 0.51 | | | |
| unconstrained variation [%] | 89.5 | | | | 95.1 | | | | 93.2 | | | | 78.2 | | | |
| constrained variation [%] | 10.5 | | | | 4.9 | | | | 6.8 | | | | 21.8 | | | |
| stepwise-built models | | | | | | | | | | | | | | | | |
| variables included in a model | legumes | 1.11 | 1.82 | 0.005 | HEI | 1.23 | 2.00 | 0.001 | frequency of defecation | 1.23 | 1.89 | 0.001 | legumes | 1.07 | 1.66 | 0.011 |
| | fruit | 1.21 | 1.83 | 0.029 | | | | | Bristol stool scale | 0.87 | 1.34 | 0.023 | fruit | 0.98 | 1.53 | 0.069 |
| | low-fat dairy products | 0.81 | 1.33 | 0.034 | | | | | | | | | HEI | 1.06 | 1.65 | 0.002 |
| | | | | | | | | | | | | | frequency of defecation | 1.31 | 2.04 | 0.002 |
| RDA1 | | 1.28 | 2.10 | 0.014 | | 1.23 | 2.00 | 0.001 | | 1.50 | 2.31 | 0.001 | | 1.49 | 2.33 | 0.007 |
| RDA2 | | 1.06 | 1.73 | 0.017 | | — | — | — | | 0.73 | 1.12 | 0.197 | | 1.35 | 2.10 | 0.005 |
| p (whole model) | 0.001 | | | | 0.001 | | | | 0.001 | | | | 0.001 | | | |
| $R^2$ (adjusted) [%] | 1.04 | | | | 0.50 | | | | 0.76 | | | | 1.77 | | | |
| unconstrained variation [%] | 97.5 | | | | 99.0 | | | | 98.2 | | | | 96.1 | | | |
| constrained variation [%] | 2.5 | | | | 1.0 | | | | 1.8 | | | | 3.9 | | | |

[1] Model 1 included the intake

[1] Model 1 included the intake of refined bread, wholegrain bread, refined cereals and groats, wholegrain cereals and groats, plant fats, animal fats, low-fat dairy products, high-fat dairy products, added sugar, soft drinks, confectionery, savory snacks, vegetables, vegetable juice, fruit, fruit juice, red meat, white meat and fish, nuts and seeds, legumes, and alcohol (as portions).

[2] Model 2 included the intake of simple carbohydrates [%E], fiber, protein [%E], SFA [%E], PUFA [%E], alcohol [%E], salt, and HEI (total carbohydrates and fat were excluded from the analysis because of collinearity)

[3] Model 3 included age, sex, body fat, WHR, physical activity level, smoking, Bristol stool scale, frequency of defecation, sudden bowel movement, constipation, diarrhea, laxative use (waist circumference, hip circumference and BMI were excluded from the analysis because of collinearity)

[4] Model 4 included the intake of refined bread, wholegrain bread, refined cereals and groats, wholegrain cereals and groats, plant fats, animal fats, low-fat dairy products, high-fat dairy products, added sugar, soft drinks, confectionery, savory snacks, vegetables, vegetable juice, fruit, fruit juice, red meat, white meat and fish, nuts and seeds, legumes, simple carbohydrates [%E], fiber, protein [%E], SFA [%E], PUFA [%E], alcohol [%E], salt, HEI, age, sex, body fat, WHR, physical activity level, smoking, Bristol stool scale, frequency of defecation, sudden bowel movement, constipation, diarrhea, laxative use (portions of alcohol were excluded, since alcohol expressed in %E was included; total carbohydrates, fat intake, waist circumference, hip circumference, and BMI were excluded from the analysis because of collinearity)
