# Supplementary material for: Human gut microbiota composition and its predicted functional properties in people with western and healthy dietary patterns
Source: Eur J Nutr. 2022 Jun 24;61(8):3887–903. doi: 10.1007/s00394-022-02928-6 (PMC9596542; doi:10.1007/s00394-022-02928-6)
Supplement: Supplementary file 5 — Supplementary file5 (PDF 107 KB) [file 394_2022_2928_MOESM5_ESM.pdf]

Supplementary Resource 5. RDA models explaining the variance in predicted metabolic pathway diversity. The results of the statistical analyses for the tested factors corresponds to the marginal term analysis in a model with all other variables included.

	Model 1, using food products[1]			Model 2, using food components and HEI[2]			Model 3, using anthropometric, lifestyle, and stool transit time data[3]			Model 4, using all variables from the other analyses[4]		
parameters of the model	variance [%]	F	p	variance [%]	F	p	variance [%]	F	p	variance [%]	F	p
RDA1	8.32	4.83	0.671	7.61	4.49	0.037	8.53	4.69	0.121	18.69	10.27	0.322
RDA2	6.37	3.70	0.748	2.98	1.76	0.836	4.42	2.43	0.821	9.39	5.16	0.990
p (whole model)	0.413			0.006			0.097			0.264		
R^2 (adjusted) [%]	0.14			1.63			0.95			0.79		
unconstrained variation [%]	89.3			94.4			92.7			78.0		
constrained variation [%]	10.7			5.6			7.3			22.0		
stepwise-built models												
variables included in a model	added sugar 4.68	2.76	0.006	HEI 4.82	2.83	0.005	frequency of defecation 4.95	2.72	0.005	legumes 3.28	1.84	0.044
	legumes 2.77	1.63	0.071	simple carb. [%E] 3.88	2.28	0.009				soft drinks 2.57	1.44	0.13
	portions of alcohol 3.59	2.11	0.016	SFA [%E] 2.44	1.43	0.109				HEI 4.87	2.74	0.002
										simple carb. [%E] 4.27	2.40	0.011
										salt 2.87	1.61	0.063
										frequency of defecation 4.49	2.52	0.008
RDA1	5.59	3.30	0.015	6.07	3.57	0.005	4.95	2.72	0.006	10.97	6.16	0.001
RDA2	2.63	1.55	0.273	1.95	1.15	0.565	—	—	—	3.39	1.90	0.503
p (whole model)	0.002			0.002			0.008			0.001		
R^2 (adjusted)	1.59			1.24			0.91			2.91		
unconstrained variation [%]	96.9			97.3			98.6			94.0		

constrained variation [%]	3.1	2.7	1.4	6.0

[1] Model 1 included the intake of refined bread, wholegrain bread, refined cereals and groats, wholegrain cereals and groats, plant fats, animal fats, low-fat dairy products, high-fat dairy products, added sugar, soft drinks, confectionery, savory snacks, vegetables, vegetable juice, fruit, fruit juice, red meat, white meat and fish, nuts and seeds, legumes, and alcohol (as portions).

[2] Model 2 included the intake of simple carbohydrates, fiber, protein, SFA, PUFA, alcohol, salt and HEI (total carbohydrates and fat were excluded from the analysis because of collinearity)

[3] Model 3 included age, sex, body fat, WHR, physical activity level, smoking, Bristol stool scale, frequency of defecation, sudden bowel movement, constipation, diarrhea, laxative use (waist circumference, hip circumference and BMI were excluded from the analysis because of collinearity)

[4] Model 4 included the intake of refined bread, wholegrain bread, refined cereals and groats, wholegrain cereals and groats, plant fats, animal fats, low-fat dairy products, high-fat dairy products, added sugar, soft drinks, confectionery, savory snacks, vegetables, vegetable juice, fruit, fruit juice, red meat, white meat and fish, nuts and seeds, legumes, simple carbohydrates [%E], fiber, protein [%E], SFA [%E], PUFA [%E], alcohol [%E], salt, HEI, age, sex, body fat, WHR, physical activity level, smoking, Bristol stool scale, frequency of defecation, sudden bowel movement, constipation, diarrhea, laxative use (portions of alcohol were excluded, since alcohol expressed in %E was included; total carbohydrates, fat intake, waist circumference, hip circumference, and BMI were excluded from the analysis because of collinearity)
